# Hospital Performance Trends on National Quality Measures and the Association With Joint Commission Accreditation

**DOI:** 10.1002/jhm.905

**Published:** 2011-10

**Authors:** Stephen P Schmaltz, Scott C Williams, Mark R Chassin, Jerod M Loeb, Robert M Wachter

**Affiliations:** 1 The Joint CommissionOakbrook Terrace, Illinois; 2 University of CaliforniaSan Francisco, San Francisco, California

## Abstract

**BACKGROUND:**

Evaluations of the impact of hospital accreditation have been previously hampered by the lack of nationally standardized data. One way to assess this impact is to compare accreditation status with other evidence-based measures of quality, such as the process measures now publicly reported by The Joint Commission and the Centers for Medicare and Medicaid Services (CMS).

**OBJECTIVES:**

To examine the association between Joint Commission accreditation status and both absolute measures of, and trends in, hospital performance on publicly reported quality measures for common diseases.

**DESIGN, SETTING, AND PATIENTS:**

Performance data for 2004 and 2008 from U.S. acute care and critical access hospitals were obtained using publicly available CMS Hospital Compare data augmented with Joint Commission performance data.

**MEASUREMENTS:**

Changes in hospital performance between 2004 and 2008, and percent of hospitals with 2008 performance exceeding 90% for 16 measures of quality-of-care and 4 summary scores.

**RESULTS:**

Hospitals accredited by The Joint Commission tended to have better baseline performance in 2004 than non-accredited hospitals. Accredited hospitals had larger gains over time, and were significantly more likely to have high performance in 2008 on 13 out of 16 standardized clinical performance measures and all summary scores.

**CONCLUSIONS:**

While Joint Commission-accredited hospitals already outperformed non-accredited hospitals on publicly reported quality measures in the early days of public reporting, these differences became significantly more pronounced over 5 years of observation. Future research should examine whether accreditation actually promotes improved performance or is a marker for other hospital characteristics associated with such performance. **Journal of Hospital Medicine** 2011;6:458–465. © 2011 Society of Hospital Medicine

The Joint Commission (TJC) currently accredits approximately 4546 acute care, critical access, and specialty hospitals,^1^ accounting for approximately 82% of U.S. hospitals (representing 92% of hospital beds). Hospitals seeking to earn and maintain accreditation undergo unannounced on-site visits by a team of Joint Commission surveyors at least once every 3 years. These surveys address a variety of domains, including the environment of care, infection prevention and control, information management, adherence to a series of national patient safety goals, and leadership.^1^

The survey process has changed markedly in recent years. Since 2002, accredited hospitals have been required to continuously collect and submit selected performance measure data to The Joint Commission throughout the three-year accreditation cycle. The tracer methodology, an evaluation method in which surveyors select a patient to follow through the organization in order to assess compliance with selected standards, was instituted in 2004. Soon thereafter, on-site surveys went from announced to unannounced in 2006.

Despite the 50+ year history of hospital accreditation in the United States, there has been surprisingly little research on the link between accreditation status and measures of hospital quality (both processes and outcomes). It is only recently that a growing number of studies have attempted to examine this relationship. Empirical support for the relationship between accreditation and other quality measures is emerging. Accredited hospitals have been shown to provide better emergency response planning^2^ and training^3^ compared to non-accredited hospitals. Accreditation has been observed to be a key predictor of patient safety system implementation^4^ and the primary driver of hospitals' patient-safety initiatives.^5^ Accredited trauma centers have been associated with significant reductions in patient mortality,^6^ and accreditation has been linked to better compliance with evidence-based methadone and substance abuse treatment.^7^, ^8^ Accredited hospitals have been shown to perform better on measures of hospital quality in acute myocardial infarction (AMI), heart failure, and pneumonia care.^9^, ^10^ Similarly, accreditation has been associated with lower risk-adjusted in-hospital mortality rates for congestive heart failure (CHF), stroke, and pneumonia.^11^, ^12^ The results of such research, however, have not always been consistent. Several studies have been unable to demonstrate a relationship between accreditation and quality measures. A study of financial and cost-related outcome measures found no relationship to accreditation,^13^ and a study comparing medication error rates across different types of organizations found no relationship to accreditation status.^14^ Similarly, a comparison of accredited versus non-accredited ambulatory surgical organizations found that patients were less likely to be hospitalized when treated at an accredited facility for colonoscopy procedures, but no such relationship was observed for the other 4 procedures studied.^15^

While the research to date has been generally supportive of the link between accreditation and other measures of health care quality, the studies were typically limited to only a few measures and/or involved relatively small samples of accredited and non-accredited organizations. Over the last decade, however, changes in the performance measurement landscape have created previously unavailable opportunities to more robustly examine the relationship between accreditation and other indicators of hospital quality.

At about the same time that The Joint Commission's accreditation process was becoming more vigorous, the Centers for Medicare and Medicaid Services (CMS) began a program of publicly reporting quality data (http://www.hospitalcompare.hhs.gov). The alignment of Joint Commission and CMS quality measures establishes a mechanism through which accredited and non-accredited hospitals can be compared using the same nationally standardized quality measures. Therefore, we took advantage of this unique circumstance—a new and more robust TJC accreditation program and the launching of public quality reporting—to examine the relationship between Joint Commission accreditation status and publicly reported hospital quality measures. Moreover, by examining trends in these publicly reported measures over five years and incorporating performance data not found in the Hospital Compare Database, we assessed whether accreditation status was also linked to the pace of performance improvement over time.

By using a population of hospitals and a range of standardized quality measures greater than those used in previous studies, we seek to address the following questions: Is Joint Commission accreditation status truly associated with higher quality care? And does accreditation status help identify hospitals that are more likely to improve their quality and safety over time?

## METHODS

### Performance Measures

Since July 2002, U.S. hospitals have been collecting data on standardized measures of quality developed by The Joint Commission and CMS. These measures have been endorsed by the National Quality Forum^16^ and adopted by the Hospital Quality Alliance.^17^ The first peer-reviewed reports using The Joint Commission/CMS measure data confirmed that the measures could successfully monitor and track hospital improvement and identify disparities in performance,^18^, ^19^ as called for by the Institute of Medicine's (IOM) landmark 2001 report, *Crossing the Quality Chasm*.^20^

In order to promote transparency in health care, both CMS—through the efforts of the Hospital Quality Alliance—and The Joint Commission began publicly reporting measure rates in 2004 using identical measure and data element specifications. It is important to note that during the five-year span covered by this study, both The Joint Commission and CMS emphasized the reporting of performance measure data. While performance improvement has been the clear objective of these efforts, neither organization established targets for measure rates or set benchmarks for performance improvement. Similarly, while Joint Commission-accredited hospitals were required to submit performance measure data as a condition of accreditation, their actual performance on the measure rates did not factor into the accreditation decision. In the absence of such direct leverage, it is interesting to note that several studies have demonstrated the positive impact of public reporting on hospital performance,^21^ and on providing useful information to the general public and health care professionals regarding hospital quality.^22^

The 16 measures used in this study address hospital compliance with evidence-based processes of care recommended by the clinical treatment guidelines of respected professional societies.^23^ Process of care measures are particularly well suited for quality improvement purposes, as they can identify deficiencies which can be immediately addressed by hospitals and do not require risk-adjustment, as opposed to outcome measures, which do not necessarily directly identify obvious performance improvement opportunities.^24^–^26^ The measures were also implemented in sets in order to provide hospitals with a more complete portrayal of quality than might be provided using unrelated individual measures. Research has demonstrated that greater collective performance on these process measures is associated with improved one-year survival after heart failure hospitalization^27^ and inpatient mortality for those Medicare patients discharged with acute myocardial infarction, heart failure, and pneumonia,^28^ while other research has shown little association with short-term outcomes.^29^

Using the Specifications Manual for National Hospital Inpatient Quality Measures,^16^ hospitals identify the initial measure populations through International Classification of Diseases (ICD-CM-9) codes and patient age obtained through administrative data. Trained abstractors then collect the data for measure-specific data elements through medical record review on the identified measure population or a sample of this population. Measure algorithms then identify patients in the numerator and denominator of each measure.

Process measure rates reflect the number of times a hospital treated a patient in a manner consistent with specific evidence-based clinical practice guidelines (numerator cases), divided by the number of patients who were eligible to receive such care (denominator cases). Because precise measure specifications permit the exclusion of patients contraindicated to receive the specific process of care for the measure, ideal performance should be characterized by measure rates that approach 100% (although rare or unpredictable situations, and the reality that no measure is perfect in its design, make consistent performance at 100% improbable). Accuracy of the measure data, as measured by data element agreement rates on reabstraction, has been reported to exceed 90%.^30^

In addition to the individual performance measures, hospital performance was assessed using 3 condition-specific summary scores, one for each of the 3 clinical areas: acute myocardial infarction, heart failure, and pneumonia. The summary scores are a weighted average of the individual measure rates in the clinical area, where the weights are the sample sizes for each of the measures.^31^ A summary score was also calculated based on all 16 measures as a summary measure of overall compliance with recommended care.

One way of studying performance measurement in a way that relates to standards is to evaluate whether a hospital achieves a high rate of performance, where high is defined as a performance rate of 90% or more. In this context, measures were created from each of the 2004 and 2008 hospital performance rates by dichotomizing them as being either less than 90%, or greater than or equal to 90%.^32^

### Data Sources

The data for the measures included in the study are available on the CMS Hospital Compare public databases or The Joint Commission for discharges in 2004 and 2008.^33^ These 16 measures, active for all 5 years of the study period, include: 7 measures related to acute myocardial infarction care; 4 measures related to heart failure care; and 5 measures related to pneumonia care. The majority of the performance data for the study were obtained from the yearly CMS Hospital Compare public download databases (http://www.medicare.gov/Download/DownloadDB.asp). When hospitals only reported to The Joint Commission (154 hospitals; of which 118 are Veterans Administration and 30 are Department of Defense hospitals), data were obtained from The Joint Commission's ORYX database, which is available for public download on The Joint Commission's Quality Check web site.^23^ Most accredited hospitals participated in Hospital Compare (95.5% of accredited hospitals in 2004 and 93.3% in 2008).

### Hospital Characteristics

We then linked the CMS performance data, augmented by The Joint Commission performance data when necessary, to hospital characteristics data in the American Hospital Association (AHA) Annual Survey with respect to profit status, number of beds (<100 beds, 100–299 beds, 300+ beds), rural status, geographic region, and whether or not the hospital was a critical access hospital. (Teaching status, although available in the AHA database, was not used in the analysis, as almost all teaching hospitals are Joint Commission accredited.) These characteristics were chosen since previous research has identified them as being associated with hospital quality.^9^, ^19^, ^34^–^37^ Data on accreditation status were obtained from The Joint Commission's hospital accreditation database. Hospitals were grouped into 3 hospital accreditation strata based on longitudinal hospital accreditation status between 2004 and 2008: 1) hospitals not accredited in the study period; 2) hospitals accredited between one to four years; and 3) hospitals accredited for the entire study period. Analyses of this middle group (those hospitals accredited for part of the study period; n = 212, 5.4% of the whole sample) led to no significant change in our findings (their performance tended to be midway between always accredited and never-accredited hospitals) and are thus omitted from our results. Instead, we present only hospitals who were never accredited (n = 762) and those who were accredited through the entire study period (n = 2917).

### Statistical Analysis

We assessed the relationship between hospital characteristics and 2004 performance of Joint Commission-accredited hospitals with hospitals that were not Joint Commission accredited using χ^2^ tests for categorical variables and *t* tests for continuous variables. Linear regression was used to estimate the five-year change in performance at each hospital as a function of accreditation group, controlling for hospital characteristics. Baseline hospital performance was also included in the regression models to control for ceiling effects for those hospitals with high baseline performance. To summarize the results, we used the regression models to calculate adjusted change in performance for each accreditation group, and calculated a 95% confidence interval and *P* value for the difference between the adjusted change scores, using bootstrap methods.^38^

Next we analyzed the association between accreditation and the likelihood of high 2008 hospital performance by dichotomizing the hospital rates, using a 90% cut point, and using logistic regression to estimate the probability of high performance as a function of accreditation group, controlling for hospital characteristics and baseline hospital performance. The logistic models were then used to calculate adjusted rates of high performance for each accreditation group in presenting the results.

We used two-sided tests for significance; *P* < 0.05 was considered statistically significant. This study had no external funding source.

## RESULTS

For the 16 individual measures used in this study, a total of 4798 hospitals participated in Hospital Compare or reported data to The Joint Commission in 2004 or 2008. Of these, 907 were excluded because the performance data were not available for either 2004 (576 hospitals) or 2008 (331 hospitals) resulting in a missing value for the change in performance score. Therefore, 3891 hospitals (81%) were included in the final analyses. The 907 excluded hospitals were more likely to be rural (50.8% vs 17.5%), be critical access hospitals (53.9% vs 13.9%), have less than 100 beds (77.4% vs 37.6%), be government owned (34.6% vs 22.1%), be for profit (61.4% vs 49.5%), or be unaccredited (79.8% vs 45.8% in 2004; 75.6% vs 12.8% in 2008), compared with the included hospitals (*P* < 0.001 for all comparisons).

### Hospital Performance at Baseline

Joint Commission-accredited hospitals were more likely to be large, for profit, or urban, and less likely to be government owned, from the Midwest, or critical access (Table 1). Non-accredited hospitals performed more poorly than accredited hospitals on most of the publicly reported measures in 2004; the only exception is the timing of initial antibiotic therapy measure for pneumonia (Table 2).

**Table 1:** Hospital Characteristics in 2004 Stratified by Joint Commission Accreditation Status

Characteristic	Non-Accredited (n = 786)	Accredited (n = 3105)	*P* Value*
Profit status, No. (%)			<0.001
For profit	60 (7.6)	586 (18.9)	
Government	289 (36.8)	569 (18.3)	
Not for profit	437 (55.6)	1,950 (62.8)	
Census region, No. (%)			<0.001
Northeast	72 (9.2)	497 (16.0)	
Midwest	345 (43.9)	716 (23.1)	
South	248 (31.6)	1,291 (41.6)	
West	121 (15.4)	601 (19.4)	
Rural setting, No. (%)			<0.001
Rural	495 (63.0)	833 (26.8)	
Urban	291 (37.0)	2,272 (73.2)	
Bed size			<0.001
<100 beds	603 (76.7)	861 (27.7)	
100–299 beds	158 (20.1)	1,444 (46.5)	
300+ beds	25 (3.2)	800 (25.8)	
Critical access hospital status, No. (%)			<0.001
Critical access hospital	376 (47.8)	164 (5.3)	
Acute care hospital	410 (52.2)	2,941 (94.7)	

* *P* values based on χ^2^ for categorical variables.

**Table 2:** Hospital Raw Performance in 2004 and 2008, Stratified by Joint Commission Accreditation Status

	2004			2008		
		
	Non-Accredited	Accredited		Non-Accredited	Accredited	
						
Quality Measure, Mean (SD)*	(n = 786)	(n = 3105)	*P* Value†	(n = 950)	(n = 2,941)	*P* Value†
AMI						
Aspirin at admission	87.1 (20.0)	92.6 (9.4)	<0.001	88.6 (22.1)	96.0 (8.6)	<0.001
Aspirin at discharge	81.2 (26.1)	88.5 (14.9)	<0.001	87.8 (22.7)	94.8 (10.1)	<0.001
ACE inhibitor for LV dysfunction	72.1 (33.4)	76.7 (22.9)	0.010	83.2 (30.5)	92.1 (14.8)	<0.001
Beta blocker at discharge	78.2 (27.9)	87.0 (16.2)	<0.001	87.4 (23.4)	95.5 (9.9)	<0.001
Smoking cessation advice	59.6 (40.8)	74.5 (29.9)	<0.001	87.2 (29.5)	97.2 (11.3)	<0.001
PCI received within 90 min	60.3 (26.2)	60.6 (23.8)	0.946	70.1 (24.8)	77.7 (19.2)	0.006
Thrombolytic agent within 30 min	27.9 (35.5)	32.1 (32.8)	0.152	31.4 (40.7)	43.7 (40.2)	0.008
Composite AMI score	80.6 (20.3)	87.7 (10.4)	<0.001	85.8 (20.0)	94.6 (8.1)	<0.001
Heart failure						
Discharge instructions	36.8 (32.3)	49.7 (28.2)	<0.001	67.4 (29.6)	82.3 (16.4)	<0.001
Assessment of LV function	63.3 (27.6)	83.6 (14.9)	<0.001	79.6 (24.4)	95.6 (8.1)	<0.001
ACE inhibitor for LV dysfunction	70.8 (27.6)	75.7 (16.3)	<0.001	82.5 (22.7)	91.5 (9.7)	<0.001
Smoking cessation advice	57.1 (36.4)	68.6 (26.2)	<0.001	81.5 (29.9)	96.1 (10.7)	<0.001
Composite heart failure score	56.3 (24.1)	71.2 (15.6)	<0.001	75.4 (22.3)	90.4 (9.4)	<0.001
Pneumonia						
Oxygenation assessment	97.4 (7.3)	98.4 (4.0)	<0.001	99.0 (3.2)	99.7 (1.2)	<0.001
Pneumococcal vaccination	45.5 (29.0)	48.7 (26.2)	0.007	79.9 (21.3)	87.9 (12.9)	<0.001
Timing of initial antibiotic therapy	80.6 (13.1)	70.9 (14.0)	<0.001	93.4 (9.2)	93.6 (6.1)	0.525
Smoking cessation advice	56.6 (33.1)	65.7 (24.8)	<0.001	81.6 (25.1)	94.4 (11.4)	<0.001
Initial antibiotic selection	73.6 (19.6)	74.1 (13.4)	0.508	86.1 (13.8)	88.6 (8.7)	<0.001
Composite pneumonia score	77.2 (10.2)	76.6 (8.2)	0.119	90.0 (9.6)	93.6 (4.9)	<0.001
Overall composite	73.7 (10.6)	78.0 (8.7)	<0.001	86.8 (11.1)	93.3 (5.0)	<0.001

**Abbreviations**: ACE, angiotensin-converting enzyme; AMI, acute myocardial infarction; LV, left ventricular; PCI, percutaneous coronary intervention.

* Calculated as the proportion of all eligible patients who received the indicated care.

† *P* values based on *t* tests.

### Five-Year Changes in Hospital Performance

Between 2004 and 2008, Joint Commission-accredited hospitals improved their performance more than did non-accredited hospitals (Table 3). After adjustment for baseline characteristics previously shown to be associated with performance, the overall relative (absolute) difference in improvement was 26% (4.2%) (AMI score difference 67% [3.9%], CHF 48% [10.1%], and pneumonia 21% [3.7%]). Accredited hospitals improved their performance significantly more than non-accredited for 13 of the 16 individual performance measures.

**Table 3:** Performance Change and Difference in Performance Change From 2004 to 2008 by Joint Commission Accreditation Status

	Change in Performance*				
				
Characteristic	Never Accredited (n = 762)	Always Accredited (n = 2,917)	Absolute Difference, Always vs Never (95% CI)†	Relative Difference, % Always vs Never	*P* Value†
AMI					
Aspirin at admission	−1.1	2.0	3.2 (1.2–5.2)	160	0.001
Aspirin at discharge	4.7	8.0	3.2 (1.4–5.1)	40	0.008
ACE inhibitor for LV dysfunction	8.5	15.9	7.4 (3.7–11.5)	47	<0.001
Beta blocker at discharge	4.4	8.4	4.0 (2.0–6.0)	48	<0.001
Smoking cessation advice	18.6	22.4	3.7 (1.1–6.9)	17	0.012
PCI received within 90 min	6.3	13.0	6.7 (0.3–14.2)	52	0.070
Thrombolytic agent within 30 min	−0.6	5.4	6.1 (−9.5–20.4)	113	0.421
Composite AMI score	2.0	5.8	3.9 (2.2–5.5)	67	<0.001
Heart failure					
Discharge instructions	24.2	35.6	11.4 (8.7–14.0)	32	<0.001
Assessment of LV function	4.6	12.8	8.3 (6.6–10.0)	65	<0.001
ACE inhibitor for LV dysfunction	10.1	15.2	5.1 (3.5–6.8)	34	<0.001
Smoking cessation advice	20.5	26.4	6.0 (3.3–8.7)	23	<0.001
Composite heart failure score	10.8	20.9	10.1 (8.3–12.0)	48	<0.001
Pneumonia					
Oxygenation assessment	0.9	1.4	0.6 (0.3–0.9)	43	<0.001
Pneumococcal vaccination	33.4	40.9	7.5 (5.6–9.4)	18	<0.001
Timing of initial antibiotic therapy	19.2	21.1	1.9 (1.1–2.7)	9	<0.001
Smoking cessation advice	21.8	27.9	6.0 (3.8–8.3)	22	<0.001
Initial antibiotic selection	13.6	14.3	0.7 (−0.5–1.9)	5	0.293
Composite pneumonia score	13.7	17.5	3.7 (2.8–4.6)	21	<0.001
Overall composite	12.0	16.1	4.2 (3.2–5.1)	26	<0.001

**Abbreviations:** ACE angiotensin-converting enzyme; AMI, acute myocardial infarction; CI, confidence interval; LV, left ventricular; PCI, percutaneous coronary intervention.

* Performance calculated as the proportion of all eligible patients who received the indicated care. Change in performance estimated based on multivariate regression adjusting for baseline performance, profit status, bed size, rural setting, critical access hospital status, and region except for PCI received within 90 minutes and thrombolytic agent within 30 minutes which did not adjust for critical access hospital status.

† *P* values and CIs calculated based on bootstrapped standard errors.

### High Performing Hospitals in 2008

The likelihood that a hospital was a high performer in 2008 was significantly associated with Joint Commission accreditation status, with a higher proportion of accredited hospitals reaching the 90% threshold compared to never-accredited hospitals (Table 4). Accredited hospitals attained the 90% threshold significantly more often for 13 of the 16 performance measures and all four summary scores, compared to non-accredited hospitals. In 2008, 82% of Joint Commission-accredited hospitals demonstrated greater than 90% on the overall summary score, compared to 48% of never-accredited hospitals. Even after adjusting for differences among hospitals, including performance at baseline, Joint Commission-accredited hospitals were more likely than never-accredited hospitals to exceed 90% performance in 2008 (84% vs 69%).

**Table 4:** Percent of Hospitals With High Performance* in 2008 by Joint Commission Accreditation Status

	Percent of Hospitals with Performance Over 90%† Adjusted (Actual)			
			
Characteristic	Never Accredited (n = 762)	Always Accredited (n = 2,917)	Odds Ratio, Always vs Never (95% CI)†	*P* Value†
AMI				
Aspirin at admission	91.8 (71.8)	93.9 (90.7)	1.38 (1.00–1.89)	0.049
Aspirin at discharge	83.7 (69.2)	88.2 (85.1)	1.45 (1.08–1.94)	0.013
ACE inhibitor for LV dysfunction	65.1 (65.8)	77.2 (76.5)	1.81 (1.32–2.50)	<0.001
Beta blocker at discharge	84.7 (69.4)	90.9 (88.4)	1.80 (1.33–2.44)	<0.001
Smoking cessation advice	91.1 (81.3)	95.9 (94.1)	2.29 (1.31–4.01)	0.004
PCI received within 90 min	21.5 (16.2)	29.9 (29.8)	1.56 (0.71–3.40)	0.265
Thrombolytic agent within 30 min	21.4 (21.3)	22.7 (23.6)	1.08 (0.42–2.74)	0.879
Composite AMI score	80.5 (56.6)	88.2 (85.9)	1.82 (1.37–2.41)	<0.001
Heart failure				
Discharge instructions	27.0 (26.3)	38.9 (39.3)	1.72 (1.30–2.27)	<0.001
Assessment of LV function	76.2 (45.0)	89.1 (88.8)	2.54 (1.95–3.31)	<0.001
ACE inhibitor for LV dysfunction	58.0 (51.4)	67.8 (68.5)	1.52 (1.21–1.92)	<0.001
Smoking cessation advice	84.2 (62.3)	90.3 (89.2)	1.76 (1.28–2.43)	<0.001
Composite heart failure score	38.2 (27.6)	61.5 (64.6)	2.57 (2.03–3.26)	<0.001
Pneumonia				
Oxygenation assessment	100 (98.2)	100 (99.8)	4.38 (1.20–1.32)	0.025
Pneumococcal vaccination	44.1 (40.3)	57.3 (58.2)	1.70 (1.36–2.12)	<0.001
Timing of initial antibiotic therapy	74.3 (79.1)	84.2 (82.7)	1.85 (1.40–2.46)	<0.001
Smoking cessation advice	76.2 (54.6)	85.8 (84.2)	1.89 (1.42–2.51)	<0.001
Initial antibiotic selection	51.8 (47.4)	51.0 (51.8)	0.97 (0.76–1.25)	0.826
Composite pneumonia score	69.3 (59.4)	85.3 (83.9)	2.58 (2.01–3.31)	<0.001
Overall composite	69.0 (47.5)	83.8 (82.0)	2.32 (1.76–3.06)	<0.001

**Abbreviations:** ACE angiotensin-converting enzyme; AMI, acute myocardial infarction; CI, confidence interval; LV, left ventricular; PCI, percutaneous coronary intervention.

* High performance defined as performance rates of 90% or more.

† Performance calculated as the proportion of all eligible patients who received the indicated care. Percent of hospitals with performance over 90% estimated based on multivariate logistic regression adjusting for baseline performance, profit status, bed size, rural setting, critical access hospital status, and region except for PCI received within 90 minutes and thrombolytic agent within 30 minutes which did not adjust for critical access hospital status. Odds ratios, CIs, and *P* values based on the logistic regression analysis.

## DISCUSSION

While accreditation has face validity and is desired by key stakeholders, it is expensive and time consuming. Stakeholders thus are justified in seeking evidence that accreditation is associated with better quality and safety. Ideally, not only would it be associated with better performance at a single point in time, it would also be associated with the pace of improvement over time.

Our study is the first, to our knowledge, to show the association of accreditation status with improvement in the trajectory of performance over a five-year period. Taking advantage of the fact that the accreditation process changed substantially at about the same time that TJC and CMS began requiring public reporting of evidence-based quality measures, we found that hospitals accredited by The Joint Commission had had larger improvements in hospital performance from 2004 to 2008 than non-accredited hospitals, even though the former started with higher baseline performance levels. This accelerated improvement was broad-based: Accredited hospitals were more likely to achieve superior performance (greater than 90% adherence to quality measures) in 2008 on 13 of 16 nationally standardized quality-of-care measures, three clinical area summary scores, and an overall score compared to hospitals that were not accredited. These results are consistent with other studies that have looked at both process and outcome measures and accreditation.^9^–^12^

It is important to note that the observed “accreditation effect” reflects a difference between hospitals that have elected to seek one particular “self-regulatory alternative to the more restrictive and extensive public regulatory or licensure requirements” with those that have not.^39^ The non-accredited hospitals that were included in this study are not considered to be “sub-standard hospitals.” In fact, hospitals not accredited by The Joint Commission have also met the standards set by Medicare in the Conditions of Participation, and our study demonstrates that these hospitals achieved reasonably strong performance on publicly reported quality measures (86.8% adherence on the composite measure in 2008) and considerable improvement over the 5 years of public reporting (average improvement on composite measure from 2004 to 2008 of 11.8%). Moreover, there are many paths to improvement, and some non-accredited hospitals achieve stellar performance on quality measures, perhaps by embracing other methods to catalyze improvement.

That said, our data demonstrate that, on average, accredited hospitals achieve superior performance on these evidence-based quality measures, and their performance improved more strikingly over time. In interpreting these results, it is important to recognize that, while Joint Commission-accredited hospitals must report quality data, performance on these measures is not directly factored into the accreditation decision; if this were not so, one could argue that this association is a statistical tautology. As it is, we believe that the 2 measures (accreditation and publicly reported quality measures) are two independent assessments of the quality of an organization, and, while the performance measures may not be a “gold standard,” a measure of their association does provide useful information about the degree to which accreditation is linked to organizational quality.

There are several potential limitations of the current study. First, while we adjusted for most of the known hospital demographic and organizational factors associated with performance, there may be unidentified factors that are associated with both accreditation and performance. This may not be relevant to a patient or payer choosing a hospital based on accreditation status (who may not care whether accreditation is simply associated with higher quality or actually helps produce such quality), but it is relevant to policy-makers, who may weigh the value of embracing accreditation versus other maneuvers (such as pay for performance or new educational requirements) as a vehicle to promote high-quality care.

A second limitation is that the specification of the measures can change over time due to the acquisition of new clinical knowledge, which makes longitudinal comparison and tracking of results over time difficult. There were two measures that had definitional changes that had noticeable impact on longitudinal trends: the AMI measure “Primary Percutaneous Coronary Intervention (PCI) Received within 90 Minutes of Hospital Arrival” (which in 2004 and 2005 used 120 minutes as the threshold), and the pneumonia measure “Antibiotic Within 4 Hours of Arrival” (which in 2007 changed the threshold to six hours). Other changes included adding angiotensin-receptor blocker therapy (ARB) as an alternative to angiotensin-converting enzyme inhibitor (ACEI) therapy in 2005 to the AMI and heart failure measures ACEI or ARB for left ventricular dysfunction. Other less significant changes have been made to the data collection methods for other measures, which could impact the interpretation of changes in performance over time. That said, these changes influenced both accredited and non-accredited hospitals equally, and we cannot think of reasons that they would have created differential impacts.

Another limitation is that the 16 process measures provide a limited picture of hospital performance. Although the three conditions in the study account for over 15% of Medicare admissions,^19^ it is possible that non-accredited hospitals performed as well as accredited hospitals on other measures of quality that were not captured by the 16 measures. As more standardized measures are added to The Joint Commission and CMS databases, it will be possible to use the same study methodology to incorporate these additional domains.

From the original cohort of 4798 hospitals reporting in 2004 or 2008, 19% were not included in the study due to missing data in either 2004 or 2008. Almost two-thirds of the hospitals excluded from the study were missing 2004 data and, of these, 77% were critical access hospitals. The majority of these critical access hospitals (97%) were non-accredited. This is in contrast to the hospitals missing 2008 data, of which only 13% were critical access. Since reporting of data to Hospital Compare was voluntary in 2004, it appears that critical access hospitals chose to wait later to report data to Hospital Compare, compared to acute care hospitals. Since critical access hospitals tended to have lower rates, smaller sample sizes, and be non-accredited, the results of the study would be expected to slightly underestimate the difference between accredited and non-accredited hospitals.

Finally, while we have argued that the publicly reported quality measures and TJC accreditation decisions provide different lenses into the quality of a given hospital, we cannot entirely exclude the possibility that there are subtle relationships between these two methods that might be partly responsible for our findings. For example, while performance measure rates do not factor directly into the accreditation decision, it is possible that Joint Commission surveyors may be influenced by their knowledge of these rates and biased in their scoring of unrelated standards during the survey process. While we cannot rule out such biases, we are aware of no research on the subject, and have no reason to believe that such biases may have confounded the analysis.

In summary, we found that Joint Commission-accredited hospitals outperformed non-accredited hospitals on nationally standardized quality measures of AMI, heart failure, and pneumonia. The performance gap between Joint Commission-accredited and non-accredited hospitals increased over the five years of the study. Future studies should incorporate more robust and varied measures of quality as outcomes, and seek to examine the nature of the observed relationship (ie, whether accreditation is simply a marker of higher quality and more rapid improvement, or the accreditation process actually helps create these salutary outcomes).
